# Chromosome-Level Assembly of the Chinese Seabass (*Lateolabrax maculatus*) Genome

**DOI:** 10.3389/fgene.2019.00275

**Published:** 2019-04-04

**Authors:** Baohua Chen, Yun Li, Wenzhu Peng, Zhixiong Zhou, Yue Shi, Fei Pu, Xuan Luo, Lin Chen, Peng Xu

**Affiliations:** ^1^State Key Laboratory of Marine Environmental Science, College of Ocean and Earth Sciences, Xiamen University, Xiamen, China; ^2^Shenzhen Research Institute of Xiamen University, Shenzhen, China; ^3^The Key Laboratory of Mariculture, Ministry of Education, Ocean University of China, Qingdao, China; ^4^State-Province Joint Engineer Laboratory of Marine Bioproducts and Technology, College of Ocean and Earth Sciences, Xiamen University, Xiamen, China; ^5^Laboratory for Marine Biology and Biotechnology, Qingdao National Laboratory for Marine Science and Technology, Qingdao, China

**Keywords:** *Lateolabrax maculatus*, seabass, genome assembly, Hi-C, teleost genome

## Introduction

**Figure 1:**
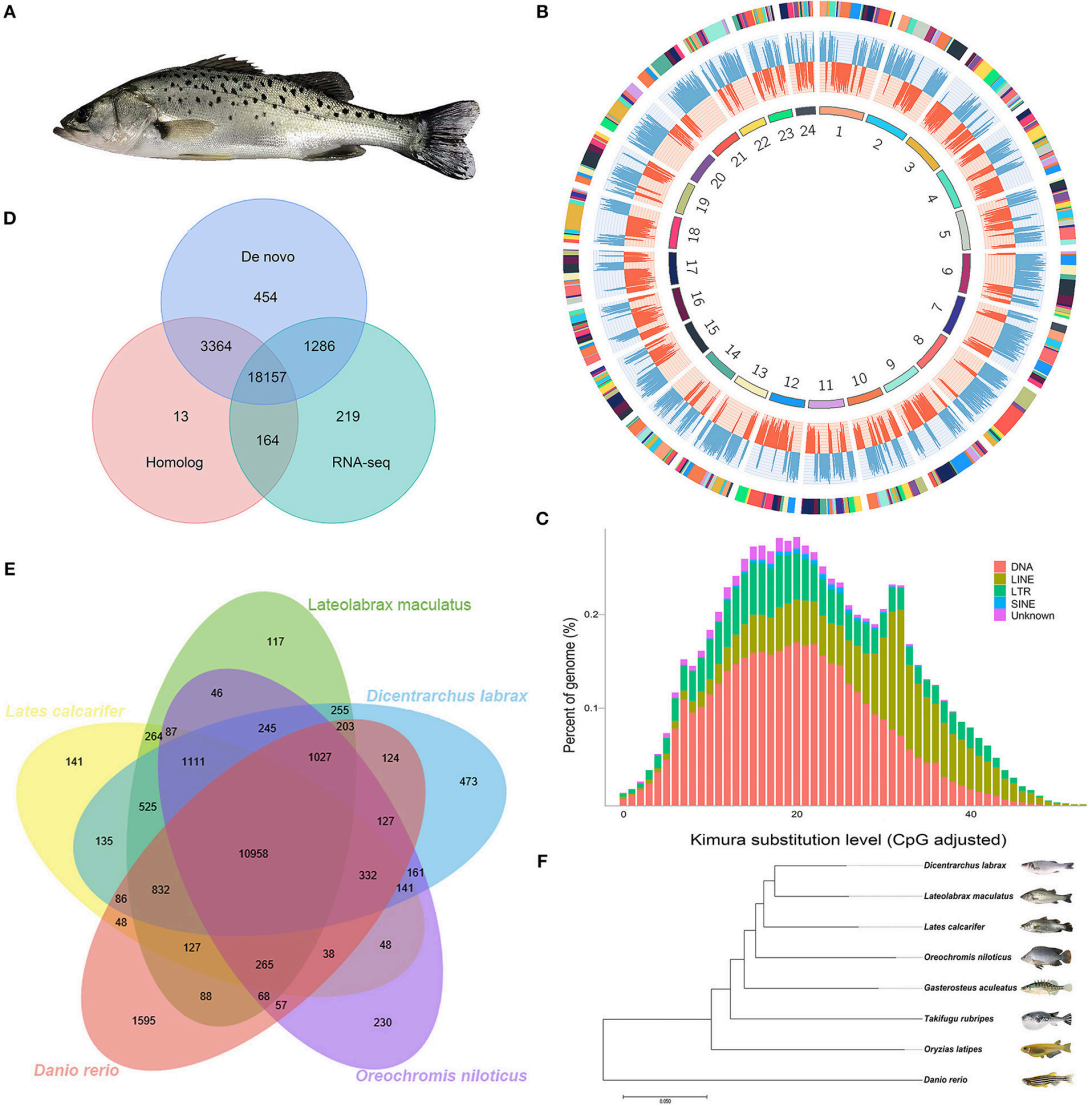
Characteristics of *Lateolabrax maculatus* genome assembly. **(A)** A picture showing about Chinese seabass, *L. maculatus*. **(B)** A circos plot of 24 chromosomes in *L. maculatus* genome, the tracks from inside to outside are: 24 chromosomes, gene abundance of the positive strand (red), gene abundance of the negative strand (blue), and scaffolds which comprised the chromosome (adjacent contigs on a chromosome are painted in different colors). **(C)** Divergence distribution of TEs in *L. maculatus* Genome. **(D)** A venn diagram indicating the number of genes predicted by three different approaches. **(E)** A venn diagram showing orthologous gene families across five fish genomes. **(F)** Evolutionary relationships among eight species.

The Chinese seabass (*Lateolabrax maculatus*), inhabiting in inshore rocky reefs and estuaries with a broad adaptability of salinity, is an euryhaline teleost fish native to the margin seas of the Northwest Pacific Ocean (Figure 1A). The Chinese seabass belongs to genus *Lateolabrax* that was first described as a geographic population of Japanese seabass *Lateolabrax japonicas*. It was recently re-described as independent species from *L. japonicas*, based on the differentiated characters of morphological traits and molecular phylogenies (Liu et al., 2006; An et al., 2014). In contrast with geographically restricted *L. japonicas, L. maculatus* is broadly and continuously distributed along the coasts of China and Indo-China Peninsula (Yokogawa and Seki, 1995). The north most of wildlife habitats of *L. maculatus* is latitude 41° north in temperate Bohai Gulf and the south most reaches at least 20° north in tropical Beibu Gulf, between which the sea surface temperature difference frequently reaches 18°C in winter. The very different environments of *L. maculatus* that live in Bohai Gulf and Beibu Gulf make them divergent in genetic structures and phenotypes, such as life history, behaviors, and breeding season etc., providing us with a feasible fish model for population genetic studies in continual marginal sea (Zhao et al., 2018). In addition, *L. maculatus* is recognized as one of the most important mariculture fish in China, which contributes over 120,000 tons of annual production. Recently, a reference genome of *L. maculatus* derived from in the northern population in Bohai Gulf had been reported (Shao et al., 2018). Herein, we report a chromosome-level genome assembly of *L. maculatus* from the southern population in the subtropical region, which provides an important resource not only for basic ecological and population genetic studies but also for the upcoming breeding program of Chinese seabass.

## Data

A whole genome shotgun (WGS) strategy was employed in this project. After removal of redundant and low-quality reads, there are a total of 112.76 Gb (188.87X) clean WGS reads, including 49.63, 31.89, 19.69, and 11.66 Gb reads from 250 bp, 2 Kbp, 5 Kbp, and 10 Kbp libraries, respectively, obtained for genome size estimation, *de novo* contig assembly, primary scaffolding. High-through chromosome conformation capture (Hi-C) sequencing were performed for chromosome-level scaffolds construction. A total of 159.54 Gb pair-end Hi-C reads were generated with an average sequencing coverage of 267.06X (Table 1).

**Table 1:** Summary of the *L. maculatus* genome assembly and annotation.

	**Bases (Gb)**	**Depth**
**SEQUENCING**		
Illumina reads (short-insert size)	49.63	83.01X
Illumina reads (long-insert size)	63.24	105.86X
Hi-C reads	159.54	267.06X
**GENOME ASSEMBLY AND CHROMOSOMES CONSTRUCTION**		
Contig N50 size (kbp)	182.31	
Contig number (>100 bp)	8719	
Scaffold N50 size (Mbp)	2.79	
Scaffold number (>100 bp)	1639	
Scaffold total length (Mbp)	597.39	
Number of chromosomes	24	
Number of placed scaffolds on chromosomes	419	
Total length of chromosomes (Mbp)	586.03	
Integration efficiency of Hi-C map (%)	98.10	
**GENOME PREDICTION AND ANNOTATION**		
Protein-coding gene number	23,657	
Mean transcript length (bp)	13,943.84	
Mean exons per gene	9.75	
Proportion of assembled CEGs (%)	94.76	
Proportion of complete BUSCO orthologs (%)	97.03	

Based on all reads mentioned above, we *de novo* assembled the draft *L. maculatus* genome with a size of 597.39 Mb containing 1,639 scaffolds. And the contig N50 size was 182.31 kb and the scaffold N50 size was 2.79 Mb. After integrating the scaffolds with Hi-C map, we finally obtained 24 chromosomes constructed from 419 scaffolds (25.56% of all scaffolds) with a total length of 586.03 Mb (98.10% of the total length of all scaffolds) (Table 1 and Figure 1B). Our new reference genome has been significantly improved compared with the previous reference genome of the northern population, which presents contig N50 length of 31 Kb, scaffold N50 length of 1,040 Kb, and chromosome integration rate of 77.68% (Shao et al., 2018).

A total of 105.5 Mb (~17.66% of *L. maculatus* genome) were identified as repetitive elements in the *L. maculatus* genome, including 6.09% of DNA transposons, 4.99% of long interspersed nuclear elements (LINEs), and 2.31% of long terminal repeats (LTRs) (Figure 1C).

Gene structure prediction identified 23,657 protein-coding genes, of which 22,509 genes can be annotated against at least one database (Figure 1D), and 1,734 candidate non-coding RNAs, including 676, 644, 99, and 315 miRNA, tRNA, rRNA, and snRNA genes, respectively (Table 1).

To evaluate the accuracy of the genome assembly, we mapped Illumina short reads that were used for genome assembly and identified 904,102 heterozygous SNPs and 12,050 false homozygous SNPs, respectively, accounting to 0.1557 and 0.0004% of the reference genome. The homozygous SNPs were false because they refer to the SNPs that only retained one alternative allele in the Illumina short reads (homozygotes for short reads data), which was different from the reference genome. The low rate of false SNPs suggests the high accuracy of the genome assembly.

The completeness and connectivity of this assembly were accessed using both Core Eukaryotic Genes Mapping Approach (CEGMA) and Benchmarking Universal Single-Copy Orthologs (BUSCO) approaches. Two hundred and thirty-five Core Eukaryotic Genes (CEGs) out of the complete set of 248 CGEs (94.76%) were covered by the assembly and 818 out of 843 searched BUSCOs (97.03%) had been completely assembled in the draft genome, suggesting a high level of completeness and connectivity of the *de novo* assembly (Table 1).

For better use of this dataset, the evolutionary position of *L. maculatus* was accessed based on single-copy genes of *L. maculatus* and seven related species (*T. rubripes, G. aculeatus, O. latipes, D. rerio, O. niloticus, L. calcarifer*, and *D. labrax*).

The protein sequences were downloaded from the Ensembl Core database (release 90). After removing the protein sequences shorter than 50 amino acids, the set of 245,644 consensus protein sequences of the seven teleost and Chinese seabass *L. maculatus* was used to construct gene families. As a result, a total of 20,788 OrthoMCL families were built (Figure 1E) and 667 single-copy ortholog protein families in a 1:1:1 manner from all eight teleost species were used for phylogenetic analysis (Figure 1F).

## Materials and Methods

### Sample Collection, Library Construction, and Sequencing

A wild adult female Chinese seabass was collected in the Xiamen Bay, Fujian, China and used to collect blood sample. The total length and body weight of this fish were 524.6 g and 34.5 cm, respectively. Total RNA and DNA extraction were performed for whole genome sequencing and whole transcriptome sequencing following our previous studies (Jiang et al., 2014; Peng et al., 2016). Four whole-genome shotgun sequencing libraries were prepared with various insert sizes ranging from 250 bp to 10 Kbp (250 bp, 2 Kbp, 5 Kbp, 10 Kbp). The 250 bp pair-end library was constructed for *de novo* contig assembly and the other three mate-pair libraries were constructed for scaffolding contigs. Before sequencing, a quality control step was performed on Agilent 2100 Bioanalyzer (Agilent Technologies, Santa Clara, CA, USA) by evaluating the distribution of fragment length. Then libraries were sequenced using the Illumina HiSeq2500 platform with a read length of 2 × 150 bp.

High-through chromosome conformation capture (Hi-C) were performed parallelly to the Illumina sequencing. DNA samples, collected from muscle tissue, were snap frozen using liquid nitrogen for 30 min and then stored at −80°C until DNA extraction. Firstly, the DNA was fixed by formaldehyde to maintain the conformation. Then it was digested by MboI restriction enzyme and repaired by biotinylated residues to form blunt-end fragments. After *in-situ* ligation of these fragments, DNA was reverse-crosslinked and purified. Before sequencing, end repair, adaptor ligation, and polymerase chain reaction were successively performed. At last, the well-prepared Hi-C libraries were sequenced using Illumina Hiseq 2500 platform with a read length of 2 × 150 bp.

### Genome Assembly

All low-quality Illumina read pairs were filtered out if any read of the pair complies with following criteria: containing adaptor sequences; the proportion of uncertain bases (represented by “N”) exceed 10%; the proportion of low-quality base (*Q* < 5) exceed 50%. After strict filtration clean, all Illumina reads were used to generate 17-mers with a window-sliding-like method. Obviously, there were 4^17^ kinds of different 17-mers. After calculation of depth distribution of these 17-mers using Jellyfish (Marcais and Kingsford, 2011) (version 2.2.5), we can estimate the genome size using Lander/Waterman's equations:

(1) $$\begin{array}{lll} {\text{C}}_{\text{base}} & = & {\text{C}}_{17-\text{mer}}\times \text{L}/\left(\text{L}-17+1\right) \end{array}$$

(2) $$\begin{array}{lll} {\text{G}}_{\text{est}} & = & {\text{N}}_{17-\text{mer}}/{\text{C}}_{17-\text{mer}}={\text{N}}_{\text{base}}/{\text{C}}_{\text{base}} \end{array}$$

In these equations, L was the length of reads (150 for Illumina reads), N_base_ and N_17−mer_ were counts of bases and 17-mers; C_base_ and C_k−mer_ were expectations of coverage depth of bases and 17-mers; estimated genome size was represented by G_est_. The genome size of *Latiolabrax maculatus* was then estimated to contain 641.02 Mb, which is similar to Asian seabass (*Lates calcarifer*, 668.5 Mb) (Vij et al., 2016) and European seabass (*Dicentrarchus labrax*, 675 Mb) (Tine et al., 2014).

For *de novo* genome assembly, high quality reads from the short-insert library (250 bp) were collected and assembled using SOAPdenovo2 (Luo et al., 2012) with optimized parameters to build initial contigs. Long-insert reads were then mapped onto the *de novo* assembled contigs for scaffolding (2, 5, and 10 Kbp, in turn). The GapCloser (Luo et al., 2012) was then used to close the gaps in scaffolds using the pair-end reads, of which one end uniquely mapped to a contig and another was located within a gap.

In order to obtain chromosome-level genome assembly, Hi-C reads were filtered in the same way as short-insert library reads and subsequently mapped to *de novo* assembled scaffolds to construct contacts among scaffolds using bwa (Li and Durbin, 2009) (version 0.7.17) with default parameters. Obtained BAM files containing Hi-C read-pairs linking messages were processed by another round of filtering, in which reads located further than 500 bp from the nearest restriction enzyme site were removed. Then LACHESIS (Korbel and Lee, 2013) (version 2e27abb) was used to chromosome-level scaffolding by clustering, ordering and orientating the *de novo* genome assemblies based on genomics proximity messages between Hi-C reads pairs. In these steps, all parameters were set as default except that CLUSTER_N, CLUSTER_MIN_RE_SITES and ORDER_MIN_N_RES_IN_SHREDS were set as 24, 80, and 10 separately. Note that the parameter CLUSTER_N was used to specify the number of chromosomes.

Both karyotype analysis and recently published genome assembly for Chinese seabass (spotted seabass) indicated that the number of chromosomes of this species is 24 (Sola et al., 1993; Shao et al., 2018). Besides, genetics maps of two species in Perciformes, European seabass (*Dicentrarchus labrax*) and Asian Seabass (*Lates calcarifer*), both contain 24 linkage groups (Wang et al., 2011; Tine et al., 2014).

### Repetitive Elements Characterization

We employed two approaches to detect repeat sequences in *L. maculatus* genome. Firstly, we used Tandem Repeats Finder (Benson, 1999) (version 4.04), Piler (Edgar and Myers, 2005) (version 1.0), LTR_FINDER (Xu and Wang, 2007) (version 1.0.2), RepeatModeler (Tarailo-Graovac and Chen, 2009) (version 1.04), and RepeatScout (Price et al., 2005) (version 1.0.2) synchronously to detect various kinds of repeat sequences in *L. maculatus* genome. The results were then combined as a single *de novo* repeat sequence library by Uclust (Edgar, 2010) (version 1.2.22q). Subsequently, the whole library was annotated using RepeatMasker (Tarailo-Graovac and Chen, 2009) (version 3.2.9) based on Repbase TE (Jurka et al., 2005) (version 14.04) to discriminate between known and novel transposable elements (TEs). In another approach, generated genome sequences were mapped on Repbase TE (Jurka et al., 2005) (version 14.04) using RepeatProteinMask (Tarailo-Graovac and Chen, 2009) (version 3.2.2), a perl script included in RepeatMasker, to detect TE proteins in *L. maculatues* genome. The results of two approaches were combined and then the redundancy was removed to obtain a final Repetitive elements set.

### Gene Structure Prediction

To access a fully annotated *L. maculatus* genome, three different approaches were employed to predict protein-coding genes. *Ab intio* gene prediction was performed on repeat-masked *L. maculatus* genome assembly using Augustus (Stanke and Morgenstern, 2005) (version 2.5.5), GlimmerHMM (Majoros et al., 2004) (version 3.0.1), SNAP (Korf, 2004) (version 1.0), Geneid (Parra et al., 2000) (version 1.4.4), and GenScan (Burge and Karlin, 1997) (version 1.0). Furthermore, homology-based prediction was performed using downloaded protein sequences of closely related teleost including *Takifugu rubripes* (Aparicio et al., 2002), *Gasterosteus aculeatus* (Jones et al., 2012), *Oryzias latipes* (Kasahara et al., 2007), *Danio rerio* (Howe et al., 2013), *Oreochromis niloticus* (Brawand et al., 2014), *Lates calcarifer* (Vij et al., 2016), *Larimichthys crocea* (Ao et al., 2015), and *Cynoglossus semilaevis* (Chen et al., 2014). Subsequently, these protein sequences were mapped onto the generated assembly using blat (Kent, 2002) (version 35) with *e*-value ≤1e-5. GeneWise (Birney et al., 2004) (version 2.2.0) was employed to align the homologs in *L. maculatus* genome against the other species for gene structure prediction. In addition, we also applied transcriptome-based prediction by using RNA-seq datasets of a pooled cDNA library of 12 tissues from the fish which was used for whole genome sequencing. The RNA-seq reads were mapped onto the genome assembly using TopHat (Trapnell et al., 2009) (version 1.2) software. The structures of all transcribed genes were predicted by Cufflinks (Trapnell et al., 2010) (version 2.2.1) with default parameters. The predicted gene sets generated from three approaches were then integrated to a non-redundant gene set using EvidenceModeler (Haas et al., 2008) (version 1.1.0). PASA (Haas et al., 2003) (version 2.0.2) was then used to annotate the gene structures. Aiming at identifying candidate non-coding RNA (ncRNA) genes, we aligned repeat-masked genome sequences against Rfam database (Burge et al., 2013) (version 11.0) using BLASTN to search homologs.

### Functional Annotation of Genes

Genes identified by structure prediction were subsequently functionally annotated by BLAST searches against the NCBI nr and SwissProt protein databases. Unidirectional best-hit of each *L. maculatus* gene was assigned as its homolog after discarding those with *E*-value <1 × 10^−5^ by alignments. Gene ontology (GO) annotations of genes were assigned using the InterProScan program (version 5.26) (Quevillon et al., 2005). KEGG annotation was performed against KEGG database, the KEGG Automatic Annotation Server (KAAS) (Moriya et al., 2007).

### The Completeness and Accuracy of the Assembly

The completeness and accuracy of the assembly were further assessed. We mapped Illumina short reads that were used for genome assembly using bwa (Li and Durbin, 2009) (version 0.7.17-r1188). Subsequently, BAM files containing mapping message were then piled up using samtools (Li et al., 2009) (version 1.8) to identify SNPs using thresholds of read depth >10 and quality score >20. Then, the assembly completeness was evaluated by Core Eukaryotic Genes Mapping Approach (CEGMA) (Parra et al., 2007) (version 2.3) and Benchmarking Universal Single-Copy Orthologs (BUSCO) (Simao et al., 2015) software (version 1.22) using vertebrate-specific database (vertebrata_odb9).

### Ortholog Analysis

Single-copy genes in *L. maculatus* and related species were identified based on gene families constructed from protein sequences of all species employing OrthoMCL (Li et al., 2003) and BLASTP software with default parameters. As there are no corresponding CDS sequences of Asian seabass proteins used in gene family analysis provided, the Asian seabass transcripts were translated into proteins using ORFinder (version 0.4.1). Single-copy ortholog proteins were aligned by MUSCLE (Edgar, 2004) (version 3.8.31). Subsequently, all obtained alignments were converted to their corresponding coding DNA sequences using an internal python script. A combined “supergene” was constructed from all the translated coding DNA alignments for minimum evolution (ME) phylogenetic tree construction using MEGA (Kumar et al., 2016) (Version 7.0.26).

## Ethics Statement

This study was approved by the Animal Care and Use Committee, College of Ocean and Earth Sciences, Xiamen University. The methods were carried out in accordance with approved guidelines.

## Author Contributions

PX conceived the study. BC, WP, and YL performed bioinformatics analysis. YL, FP, YS, and XL collected samples. ZZ and LC extracted DNA and RNA. ZZ and YS performed the quality control. BC and PX wrote the manuscript. All authors read and approved the final manuscript.

### Conflict of Interest Statement

The authors declare that the research was conducted in the absence of any commercial or financial relationships that could be construed as a potential conflict of interest. The reviewer GW declared a past co-authorship with one of the authors YL to the handling editor.
